# A primary neuroendocrine tumor of the left ventricle presenting with diarrhea—an unusual experience and literature review

**DOI:** 10.1186/s13000-020-00935-x

**Published:** 2020-04-03

**Authors:** Chengfang Li, Jiajia Huang, Xiaorong Yang, Jinhua Xia, Gaoqiang Xu, Hong Zheng

**Affiliations:** 1 Department of Pathology, The Affiliated Hospital of Zunyi Medical University, Zunyi, 563000 Guizhou China; 2 Department of Imaging, The Affiliated Hospital of Zunyi Medical University, Zunyi, 563000 Guizhou China

**Keywords:** Neuroendocrine tumor, Cardiac, Carcinoid syndrome, Immunohistochemistry

## Abstract

**Background:**

Neuroendocrine tumors (NETs) can secrete bioactive amines in the bloodstream, resulting in the carcinoid syndrome characterized by diarrhea and flushing. The frequency of occurrence of primary cardiac neuroendocrine neoplasms is lesser than that of metastases, and hence, metastases must be adequately ruled out before diagnosis. Cardiac tumors, both primary and metastatic, mainly result in heart-related symptoms, such as heart failure and acquired valvular dysfunction. Here, we report a unique case of a primary left ventricular neuroendocrine tumor presenting with diarrhea.

**Case presentation:**

A 51-year-old female complaining of intermittent diarrhea for 2 years was admitted to our hospital. Enhancement of total abdominal computed tomography scan, echocardiography, and magnetic resonance imaging indicated a mass in the left ventricle. The indexes of myocardial enzymes were normal. Histologically, round cells with well-differentiated neuroendocrine morphology were arranged in typical pseudo-glandular, trabecular, ribbon-like, and solid nest patterns. Immunohistochemically, the tumor cells were positive for cytokeratin, chromogranin, synaptophysin, and CD56. However, they were negative for caudal type homeobox 2, S100, paired box gene 8, thyroid transcription factor 1, and CD20, which ruled out the origin of gastrointestinal, pancreatic, lung, and Merkel cell carcinomas. The symptoms of diarrhea disappeared after the operation. The patient was asymptomatic at the 9-month follow-up.

**Conclusion:**

Cardiac neuroendocrine tumors with diarrhea are considerably rare and related clinical research is limited. We presented a case and reviewed related articles to improve the identification, diagnosis, and management of patients with cardiac neuroendocrine tumors. The site of origin of a neuroendocrine tumor is clinically vital, and identification of an occult primary tumor using imaging modalities is necessary. Immunohistochemistry is well-suited to indicate the origin of the tumor. Regular follow-up is necessary for both poorly differentiated and well-differentiated cardiac neuroendocrine tumors. It is suggested to detect some neuroendocrinal markers for patients with unexplained reasons of diarrhea.

## Background

Cardiac tumors can be either primary or metastatic. Primary tumors of the heart are exceedingly rare, with an incidence rate of 0.0017 to 0.19% in unselected autopsy series [1]. Tumors that metastasize to the heart are more common than primary tumors, similar to metastatic breast carcinomas, melanomas, lymphomas, leukemia, and sarcomas. Neuroendocrine tumors (NETs) of the heart are extremely rare, and most metastasize from gastrointestinal or pulmonary tumors. Therefore, the presence of metastatic tumors should be ruled out before the diagnosis of primary cardiac NETs. NETs can produce various biologically active substances that are capable of eliciting symptoms of the carcinoid syndrome (CS) [2] such as diarrhea and flushing. CS is the most established of all hormonal syndromes and is caused by release of 5-hydroxytryptamine (serotonin), bradykinins, and many other mediators. NET is also a cause of acquired valvular heart disease, with cardiac involvement described in up to 60% of patients with CS [3]. A population-based study showed that the frequency of occurrence of CS in patients with NET was 19%. CS is significantly associated with tumor grade, stage, and primary tumor site, and leads to shorter survival [4].

To our knowledge, patients with cardiac tumors typically present with few symptoms until the neoplasm is considerably large or has metastasized, at which point they present heart-related symptoms such as heart failure and acquired valvular dysfunction. The clinician’s challenge is to detect the tumor before the appearance of cardiac symptoms and to exclude metastatic neuroendocrine tumors from tumors of other origin using immunohistochemistry and other adjuvant examination. Here, we report the case of a patient with primary cardiac NET, which prompted a literature review on this topic.

## Case presentation

### Clinical history

**Fig. 1:**
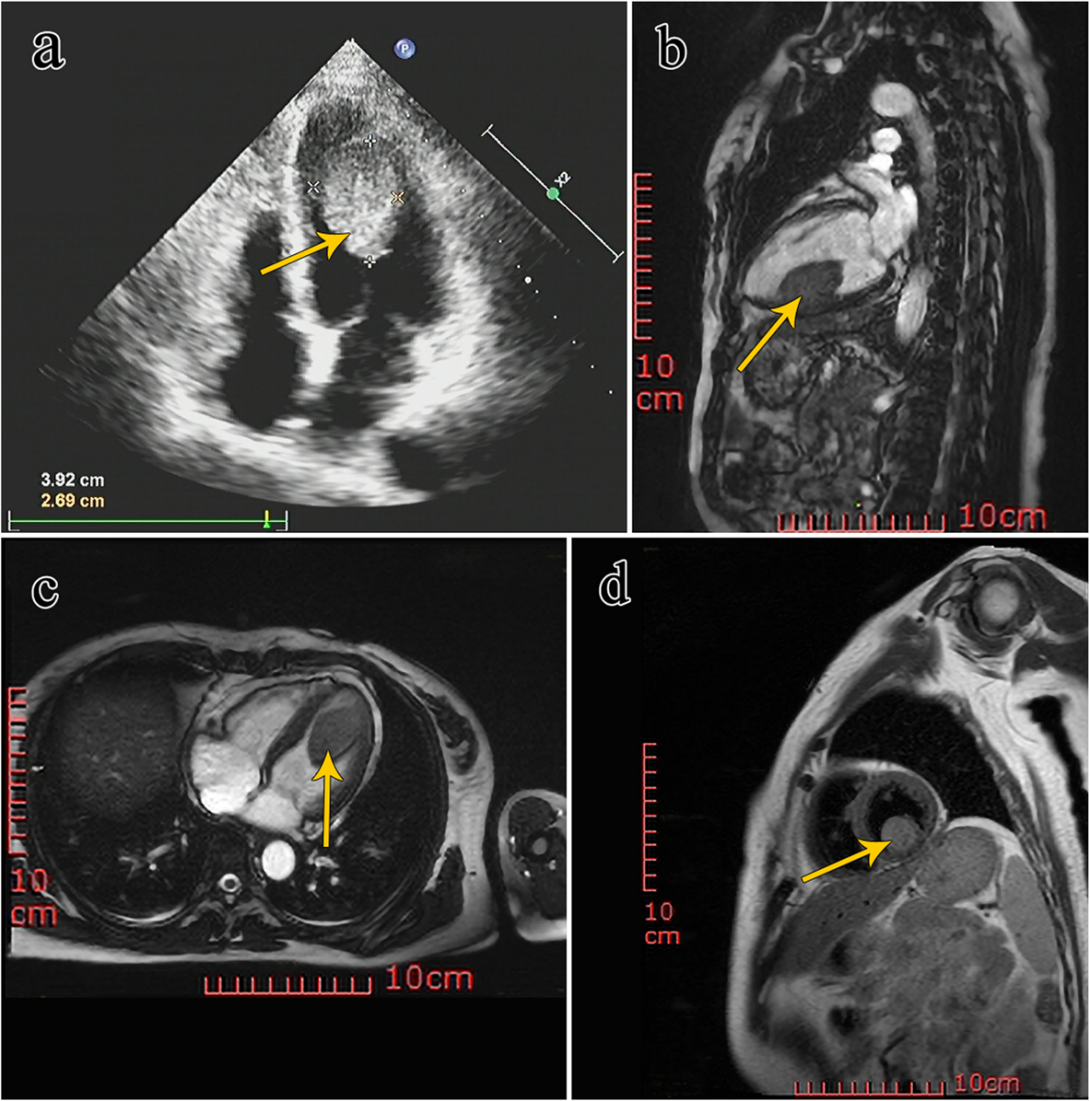
**a** Echocardiography showed a homogeneous mass in the left ventricle (the yellow arrow). **b, c** Magnetic resonance imaging (MRI) showed a longer T2 signal of the left ventricular mass (the yellow arrow). **d** MRI demonstrating round mass with clear boundary in left ventricle (the yellow arrow)

A 51-year-old female, who had been complaining of intermittent diarrhea for the previous 2 years, was admitted to our hospital with recurrent watery diarrhea along with abdominal pain for 3 days. The patient had received intermittent treatment of anti-inflammatory drugs and symptomatic treatment for 2 years due to recurrent diarrhea. However, the treatment was not effective and she had lost 5 kg body weight. To determine the specific cause of weight loss and recurrence of diarrhea, the doctor examined the patient thoroughly. No abnormality was evident in gastroscopy and proctoscopy. Enhancement of total abdominal computed tomography (CT) scan suggested a suspected space occupation in the left ventricle. Echocardiography indicated a 45 mm × 28 mm mass in the left ventricle, and a malignant tumor was suspected (Fig. 1a). Magnetic resonance imaging (MRI) was performed to confirm the diagnosis; the image showed an oval mass measuring 28 cm × 26 cm × 41 cm in the inferior wall of the left ventricle with unclear boundary and limited movement, and equal T1 and long T2 signals (Fig. 1b, c, d), and a myxoma was suspected. All the indexes of myocardial enzymes were normal. The urine metabolites were normal with exception of slightly elevated calcium levels (2.68 mmol/L, normal reference range: 2.20–2.65 mmol/L) and slightly elevated phosphorus levels (1.95 mmol/L, 0.81–1.45 mmol/L). The patient was transferred from the Department of Gastroenterology to that of Cardiac Surgery for surgical tumor resection.

### Operation

The mass was relatively isolated. Hence a median sternotomy was performed, cardiopulmonary bypass was initiated, and the heart was arrested with antegrade cardioplegia. The mass, located in the left ventricle measuring 35 mm × 25 mm, adhered to the myocardium of the diaphragmatic surface. Sharp dissection was performed circumferentially around the capsule of the mass, ensuring macroscopically free tumor margins. The heart restarted automatically when the ascending aorta was opened, although the heart rate was slow (60 times/min); it was then sewn to the pacemaker wire on the heart surface, after which it worked well. After chest closure, the patient was transferred to the cardiovascular intensive care unit for routine care. Her course was uneventful with the exception of reduction in hemoglobin and platelet levels, and slight increase in lactate dehydrogenase level.

### Pathology

Macroscopically, the lesion consisted of a 3 cm × 2 cm × 1.5 cm grayish yellow mass with a complete capsule and medium texture.

**Fig. 2:**
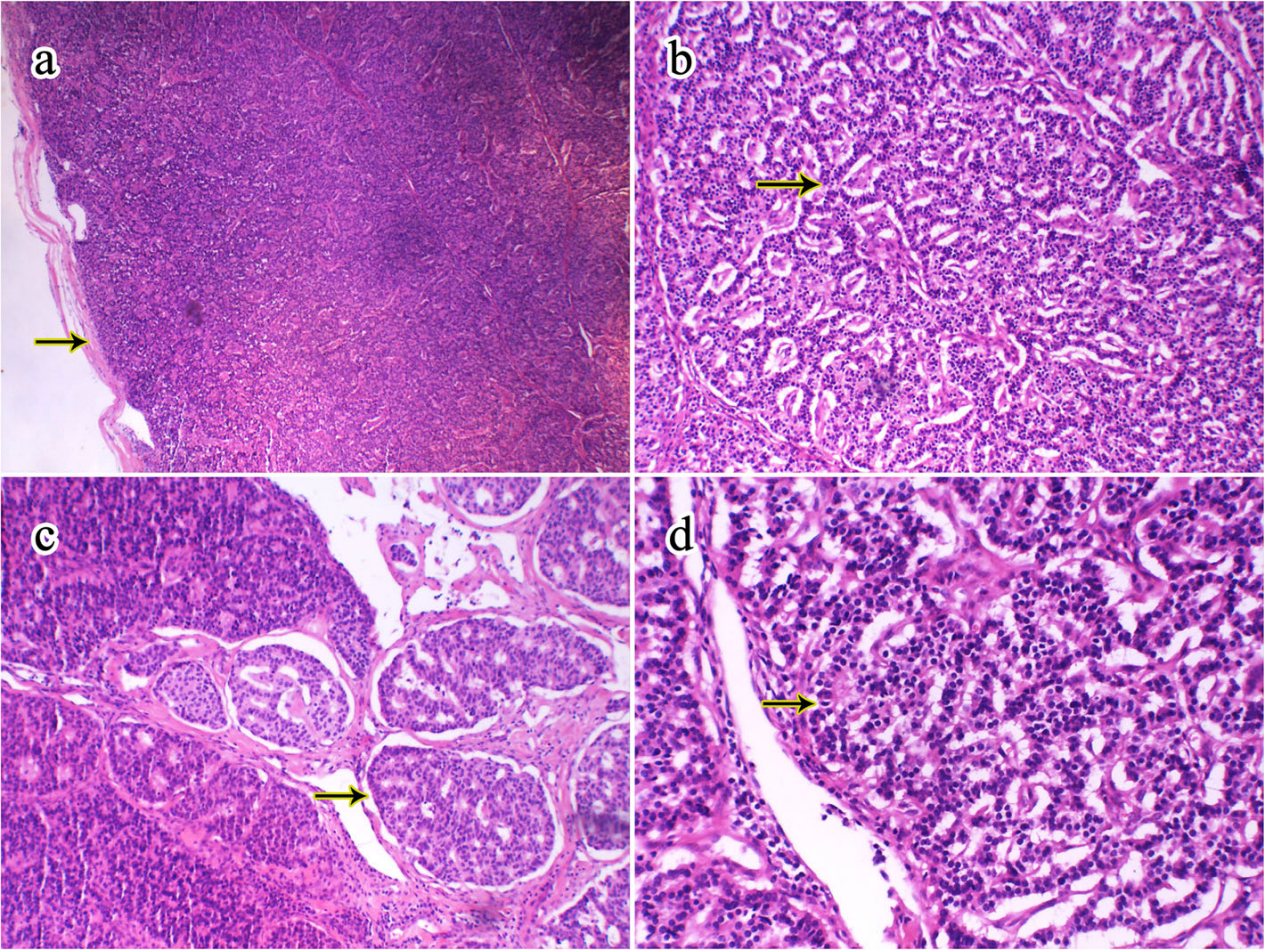
The image of HE. **a** showed a complete capsule of the mass (40×, the arrow); **b** demonstrated tumor cells arranged with pseudo-glandular (the arrow), **c** presented sieve-like patterns in tumor cells (100×). **d** The round cells were well-differentiated (200×)

Histological analysis showed that the complete capsule of the tumor was visible at low magnification (Fig. 2a). Round tumor cells of well-differentiated neuroendocrine morphology were arranged in typical pseudo-glandular, trabecular, ribbon-like, and sieve-like patterns (Fig. 2b, c). The cells were well-differentiated (Fig. 2d), no tumor necrosis was detected, and the mitotic count was 2/10HPF.

### Immunohistochemistry

**Fig. 3:**
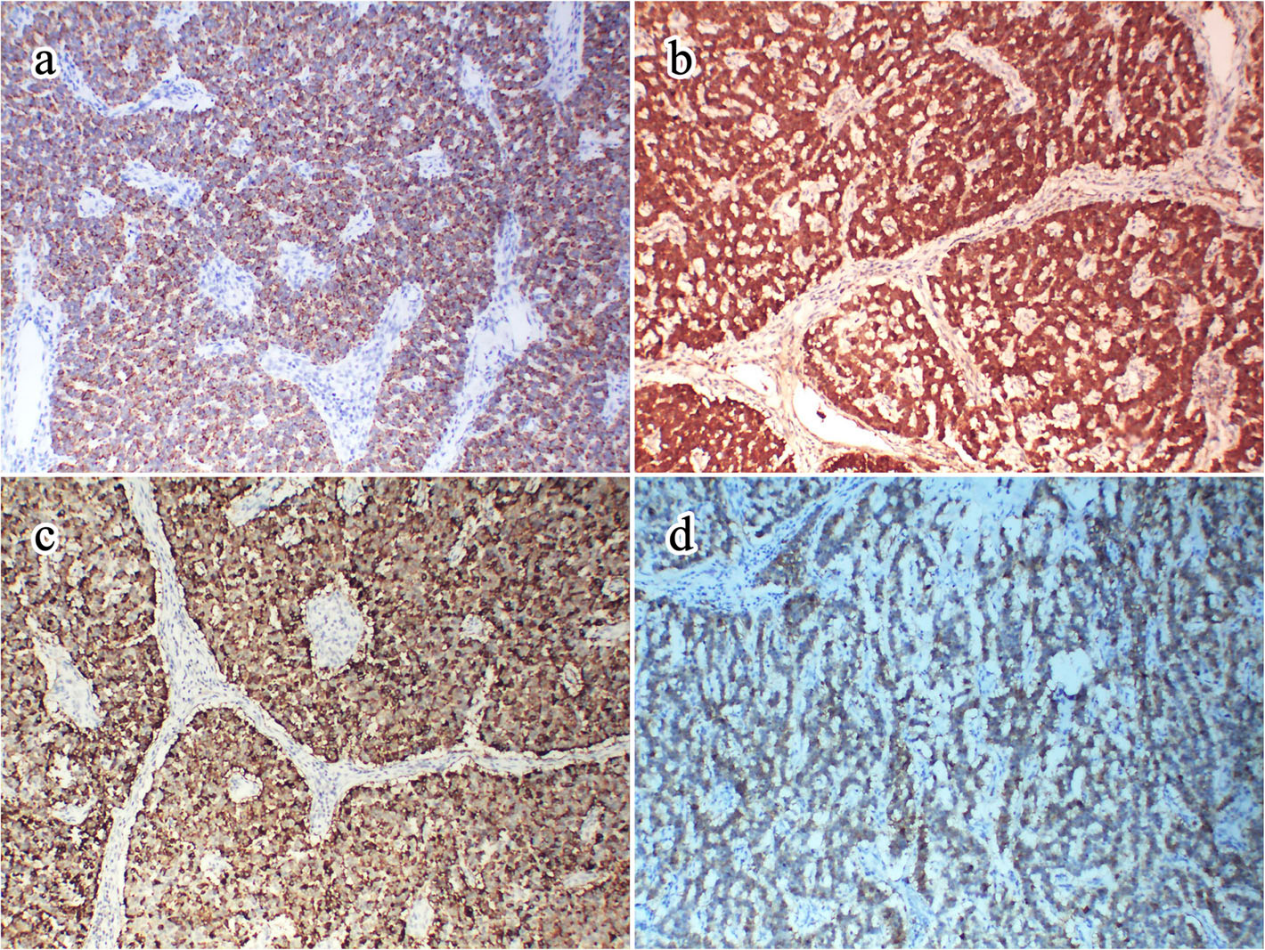
The image of immunohistochemistry. **a** represented the tumor cells were dot-like positive for CK (100×). **b**, **c** showed diffusely cytoplasmic positive for NSE, CGA respectively in tumor cells (100×). **d** Tumor cells showed patchy cytoplasmic positive for CD56 (100×)

The tumor cells were positive for cytokeratin (CK), CD56, chromogranin (CGA), neuron-specific enolase (NSE) (Fig. 3b, c, d), and synaptophysin (Syn), and were negative for vimentin and S100. The proliferative index of Ki67 was 2%. Diagnosis of neuroendocrine tumor (G1) was based on the results of immunohistochemistry and morphological analysis. Metastatic tumors were considered first because of the special anatomical location of the tumor. Further investigations with multiple imaging modalities, focusing on the appendix, gastrointestinal tract, pancreas, and lungs, did not reveal any primary tumor. Immunohistochemistry plays an important role in differential diagnosis in the absence of other tumor lesions. Hence, immunohistochemistry was performed with thyroid transcription factor 1 (TTF1) to rule out the possibility of tumor origin in the lung, with paired box gene 8 (PAX8) to rule out origin in the pancreas, with caudal type homeobox 2 (CDX2) to rule out origin in the gastrointestinal tract, and with CD20 to rule out Merkel cell carcinoma. However, the results for these makers were all negative.

The final pathological diagnosis was primary well-differentiated neuroendocrine tumor (WDNET) of the left ventricle (G1).

### Outcome and follow-up

The course of the patient was uneventful, and the symptoms of abdominal pain and diarrhea disappeared after the operation. She was discharged 10 days after the operation and returned to the hospital for reexamination at 3, 6, and 9 months post-operation. The patient was asymptomatic, with no abnormality in cardiac function and no recurrence of tumor and diarrhea.

## Discussion

Neuroendocrine neoplasms (NENs) are a heterogeneous group of epithelial neoplastic lesions that show features of neural and endocrine differentiation, including the ability to produce amines and/or peptide hormones irrespective of their primary site of origin [5]. Cardiac neuroendocrine tumors are extremely rare and are usually the result of metastatic disease. So far, only five cases of primary cardiac neuroendocrine tumors have been reported (Table 1). To the best of our knowledge, this is the first report of a patient with primary cardiac neuroendocrine tumor presenting watery diarrhea.

**Table 1:** Clinicopathologic features of primary cardiac neuroendocrine neoplasms

NO./Ref.	Sex/age	Location	Symptoms	Operation/Treatment	IHC	Histological classification	Follow-up
1 [6]	M/50	LV and RV	Chest pain	Chemotherapy+Heart transplantation	CK^+^, CD56^+^, SYN^+^, CGA^+^, P53^+^	G3	Multiple metastases 11 month post operation
2 [7]	M/54	LA	Syncope and hypotension	Local biopsy+ chemotherapy	CD99^+^, CD56^+^, CD57^+^, NSE^+^, SYN^+^, CK^+,^; S100^−^, CGA^−^, TTF1^−^	G3	Alive but multiple metastases 1 year later
3 [8]	F/68	RV	Heart failure and cyanosis	Surgical tumour resection+chemotherapy	No provide	G2	Asymptomatic at a 9-month follow-up visit
4 [9]	M/70	RA	Progressive dyspnea	Palliative surgical tumour resection+radiation therapy	CK^+^, CK20^+^, SYN^+^ vimentin^+^; CK7^−^, PSA^−^, Melan-A^−^, LCA^−^, CGA^−^	G3	Multiple metastases and died 18 months postoperatively
5 [10]	F/68	LV	Asymptomatic	Surgical tumour resection	SYN^+^, CGA^+^, CAM5.2^+^	G1	Asymptomatic at 6-month clinic follow-up
6^a^	F/51	LV	Diarrhea	Surgical tumour resection	SYN^+^, CGA^+^, CD56^+^, CK^+^, NSE^+^, Ki-67 2%; TTF1^−^, CDX2^−^, PAX-8^−^, CD20^−^, S100^−^	G1	Asymptomatic at a 9 month follow-up visit

^a^: Present case; *LV* Left ventricle, *RV* Right ventricle, *RA* Right atrium, *LA* Left atrial, *F* Female, *M* Man, *IHC* Immunohistochemistry

NETs release vasoactive peptides and amines, such as serotonin and tachykinins, resulting in CS. The most common symptoms of CS are diarrhea and flushing, and less commonly, bronchospasm and telangiectasia [11]. CS is predominantly encountered in patients with midgut NETs, the most frequently observed primary sites for NET being the small bowel, although it can also occur in patients with NETs of other origin [4]. The presence of watery diarrhea, in association with cutaneous facial flushing (especially when associated with elevated serotonin blood levels), and radiographic evidence of malignancy is almost always diagnostic of CS-associated diarrhea [12]. In the present case, the patient suffered from an unknown cause of watery diarrhea for 2 years, which resulted in high psychological and economic burden. Unexpectedly, the diarrhea disappeared after tumor resection. CS diarrhea is largely a consequence of tumoral secretion of serotonin, which results in reduced absorption of water and electrolytes, leading to diarrhea [13–15]. In our present case, serotonin blood levels were not determined because of the technical limitations of our institution, normal urine metabolites of the patient may be due to his intermittent symptomatic treatment in the local hospital. Although there is not enough laboratory data to prove that the patient had carcinoid syndrome, we highly suspected it to be CS-associated diarrhea.. The other five reported cases of cardiac neuroendocrine tumors were associated with chest pain, syncope and hypotension, heart failure and cyanosis, progressive dyspnea, and hypotension and dyspnea, respectively (Table 1). Most of the patients with cardiac neuroendocrine tumors showed local symptoms of the heart, and none suffered from diarrhea (Table 1). The symptom of diarrhea in the present case misled the clinicians, and hence the condition had not been correctly diagnosed and treated for two years. This was a case of well-differentiated neuroendocrine tumor, which was consistent with previous reports showing that CS was more frequent in patients with well-differentiated (grade I–II) tumors than advanced disease [4].

A high index of suspicion for metastatic disease in cardiac neuroendocrine tumor should prompt further investigations for identifying occult primary tumors. The most common primary sites of these tumors are the gastrointestinal tract, lungs, and mediastinum, followed by the skin (Merkel cell carcinoma) [16–19].

Certain imaging modalities such as CT and MRI might be used to assess the extended range of the tumor and diagnose primary tumors in the case of metastasis. We scanned the appendix, pancreas, lung, and gastrointestinal tract using CT but did not detect tumors of primary origin. At this stage, most pathologists may delegate the problem to the clinicians, leading to an ambiguous diagnosis.

Immunohistochemical markers are useful for identifying the origin of these tumors. Diagnostic pathologists routinely use CDX2 as a marker of intestinal-type adenocarcinomas. Two early immunohistochemical surveys reported that CDX2 expression is highly sensitive and fairly specific for neuroendocrine tumors of midgut origin (jejunoileum or appendix) [20, 21]. CDX2 shows high sensitivity and specificity for small intestinal NENs, whereas PAX8 is used to identify primary and metastatic pancreatic NETs [22]. TTF1 is most commonly used in diagnostic pathology as a marker of lung adenocarcinoma and thyroid tumors. In the setting of WDNET, TTF1 has been an incredibly specific marker for tumors of pulmonary origin, although the sensitivity varied [23–26]. A few groups have reported strong expression of PAX8 in the islets of Langerhans, which has been used to suggest the pancreatic origin of WDNET [27–29]. CK20 is normally expressed by the gastric foveolar epithelium, intestinal epithelium, urothelial umbrella cells, and Merkel cells in the skin. Typically dot-like, but sometimes diffuse CK20 expression was observed in the vast majority of Merkel cell carcinomas [30, 31]. A review mentioned that the following marker panel is recommended for suspected WDNETs: broad spectrum keratins (CK), general neuroendocrine makers, CK20, TTF1, CDX2, and PAX8 [32]. Nevertheless, subsequently study have demonstrated expression of PAX-8 in NETs of extrapancreatic origin [33]. There are no immunohistochemical makers specific for NETs of the pancreas. It should be closely combined with imaging examination at this time. We performed immunohistochemical staining using this panel in the present case, and observed that the results were negative for all with the exception of the neuroendocrine markers and CK, because of which the site of origin could not be determined and the tumor was considered primary. However, effective immunohistochemical markers for determining the origin of these tumors were not available from the other cases reviewed. As no similar report has proposed the utility of immunohistochemistry in determining the origin of cardiac neuroendocrine tumors, information in this field is limited.

As primary cardiac NET is extremely rare, and randomized trials for NET patients with solitary cardiac involvement are lacking, individual choices have to be made. In general, tumor resection is the treatment of choice, which mostly leads to positive outcomes if macroscopically free tumor margins are ensured [8, 10]. Treatment for high-grade neuroendocrine carcinoma usually consists of surgical resection and platinum-based chemotherapy [12]. Literature review indicated that alternative therapies (chemotherapy and radiation therapy) were considered when there was no possibility of resection; however, the prognosis was still poor [6, 7, 9] due to remote metastasis. On the other hand, owing to the necessity of inducing immunosuppression, heart transplantation should be avoided in patients with systemic malignant tumor [6]. The tumor, which was histologically well-differentiated, was completely removed in the present case and the patient showed good outcome.

## Conclusions

Cardiac NET is exceedingly rare and generally secondary to metastatic disease from a gastrointestinal source and other sites. The site of origin of a NET is clinically vital, and identification of occult primary tumors using imaging modalities and immunohistochemistry is necessary. Only when metastatic tumors are adequately excluded can primary cardiac NETs be diagnosed. Here, we describe the first case of primary cardiac NET presenting with diarrhea; we used imaging techniques and immunohistochemistry for determining the origin of the tumor. This type of rare tumor with unique clinical manifestation is possibly ignored by clinicians preoperatively, especially when the patient lacks typical cardiac symptoms. Furthermore, although surgical resection is the main treatment for well-differentiated cardiac NETs, early detection and correct diagnosis is undoubtedly the best option for patients. Regular follow-up is necessary for both poorly differentiated and well-differentiated cardiac NETs. It is worth noting that clinician should detect some neuroendocrinal markers for patients with unexplained reasons of diarrhea to find the cause. Finally, we expect that our case report will contribute to better recognition of these lesions and assist in avoiding inappropriate overtreatment.

## Data Availability

The data used and/or analyzed during the current study are available from the corresponding author on reasonable request.

## Abbreviations

CDX2: Caudal type homeobox 2
CGA: Chromogranin
CK: Cytokeratin
CS: Carcinoid syndrome
CT: Computed tomography
IHC: Immunohistochemistry
MRI: Magnetic resonance imaging
NEN: Neuroendocrine neoplasms
NSE: Neuron-specific enolase
PAX8: Paired box gene 8
SYN: Synaptophysin
TTF-1: Thyroid transcription factor-1
WDNET: Well differentiation neuroendocrine tumor
